# Development of a Molecular Assay for Detection and Quantification of the *BRAF* Variation in Residual Tissue From Thyroid Nodule Fine-Needle Aspiration Biopsy Specimens

**DOI:** 10.1001/jamanetworkopen.2021.27243

**Published:** 2021-10-06

**Authors:** Guodong Fu, Ronald S. Chazen, Christina MacMillan, Ian J. Witterick

**Affiliations:** 1 Alex and Simona Shnaider Research Laboratory in Molecular Oncology, Lunenfeld-Tanenbaum Research Institute, Sinai Health System, Mount Sinai Hospital, Toronto, Canada; 2 Department of Pathology and Laboratory Medicine, Sinai Health System, Mount Sinai Hospital, University of Toronto, Toronto, Canada; 3 Joseph and Mildred Sonshine Family Centre for Head and Neck Diseases, Sinai Health System, Mount Sinai Hospital, Toronto, Canada; 4 Department of Otolaryngology-Head and Neck Surgery, Sinai Health System, Mount Sinai Hospital, University of Toronto, Toronto, Canada.

## Abstract

**Importance:**

Thyroid cancer, predominantly papillary thyroid carcinoma (PTC), is common, but an estimated 30% of ultrasonography-guided fine-needle aspiration (FNA) biopsies of thyroid nodules are indeterminate. *BRAF* variation, associated with poor clinicopathological characteristics, is a useful molecular marker for diagnostics.

**Objective:**

To develop a sensitive molecular assay for BRAF V600E detection in remaining tissue of thyroid FNA biopsies to identify patients with cancer carrying a *BRAF* variation.

**Design, Setting, and Participants:**

This diagnostic study used tumor tissue from surgical formalin-fixed, paraffin-embedded (FFPE) specimens and residual tissue from thyroid FNA biopsies for genomic DNA extraction. FFPE specimens served as the validation set, and residual tissue from FNA biopsies served as the test set. A molecular assay was developed for accurate detection of BRAF V600E variation using locked nucleic acid (LNA) probe–based droplet digital polymerase chain reaction (dPCR), and the assay was validated by BRAF V600E immunohistochemical staining (IHC). The study was conducted between February 2019 and May 2021.

**Results:**

A total of 271 specimens, including 77 FFPE specimens (with a follow-up of 48 matched surgical specimens) and 146 residual FNA samples, were collected from 223 patients (mean [SD] age, 53.8 [15.3] years; 174 [78.0%] women; 49 [22.0%] men). The molecular assay by dPCR was first established to specifically and accurately detect and quantify wild-type *BRAF* and variant *BRAF* in DNA from human follicular thyroid carcinoma–derived FTC-133 and papillary thyroid carcinoma–derived BCPAP cells. The linearity of quantification of BRAF V600E was calculated (y = 0.7339x; *R*^2^ = 0.9996) with sensitivity at 0.02 copies/μL and reproducibility in detecting variant DNA at various dilutions(coefficient of variance in 0.3% DNA, 9.63%; coefficient of variance in 1.0% DNA, 7.41%). In validation testing, the dPCR assay and IHC staining exhibited 100% specificity in concordantly identifying BRAF V600E in PTCs (κ = 0.873; *P* < .001) and sensitivity of 32.0% (95% CI, 19.1% to 44.9%) in dPCR and 26.0% (95% CI, 13.1% to 38.9%) in IHC staining, with an improvement by 23.08% in dPCR compared with the IHC staining. The dPCR assay further detected BRAF V600E in 39 of 146 residual FNA specimens (26.7%). At short-term follow-up, 48 patients, including 14 of 39 patients with *BRAF* variation and 34 of 107 patients without *BRAF* variation on residual FNA specimens, underwent resection. The dPCR assay of *BRAF* status in the matched surgical specimens showed BRAF V600E variations in 12 patients and wild-type *BRAF* in 36 patients, with a high agreement to that in residual tissue of FNA specimens (κ = 0.789; *P* < .001). Among 14 patients with *BRAF* variations on residual FNA, 13 were diagnosed with PTC and 1 was diagnosed with anaplastic thyroid cancer at the thyroidectomy.

**Conclusions and Relevance:**

This diagnostic study developed a sensitive molecular assay for detection and quantification of BRAF V600E variation in residual tissue from thyroid FNA biopsies to identify patients with cancer harboring BRAF V600E in a cost-effective manner, highlighting the clinical value of molecular assay of the remaining FNA tissue in the management of thyroid nodules.

## Key Points

**Question:**

Is the residual tissue from thyroid fine-needle aspiration (FNA) biopsies clinically valuable in molecular assay to aid diagnosis of thyroid nodules with indeterminate FNA findings?

**Findings:**

This diagnostic study developed a molecular assay using digital polymerase chain reaction (dPCR) for sensitive detection and absolute quantification of BRAF V600E variation in the residual tissue from routine thyroid FNA biopsies. The dPCR testing showed better sensitivity than immunohistochemical staining and good concordance between residual tissue of FNA biopsies and matched surgical specimens.

**Meaning:**

These findings suggest that the molecular assay by dPCR can be widely implemented to detect BRAF V600E in residual FNA biopsy specimens to improve the management of thyroid nodules harboring *BRAF* variation in a cost-effective manner.

## Teaser

This diagnostic study develops and tests a sensitive molecular assay for BRAF V600E variation detection in the remaining tissue from fine-needle aspiration biopsies of thyroid nodules.

## Introduction

Thyroid nodules are detected in up to 70% of the population, while only 7% to 15% of them are malignant.^1,2^ Ultrasonography-guided fine-needle aspiration (FNA) cytological examination has served as the criterion standard for preoperative diagnosis of thyroid nodules. Each thyroid FNA is classified into 1 of 6 diagnostic categories: (1) nondiagnostic or unsatisfactory (ND), (2) benign, (3) atypia of undetermined significance/follicular lesion of undetermined significance (AUS/FLUS), (4) follicular neoplasm/suspicious for follicular neoplasm (FN/SFN), (5) suspicious for malignancy (SFM), and (6) malignant, based on the Bethesda System for Reporting Thyroid Cytopathology.^3^ Fortunately, most thyroid lesions can be correctly diagnosed as benign (70%-75%) or malignant (5%-10%). However, cytological examination cannot discriminate between benign and cancerous nodules in an estimated 30% of FNAs, categorizing them instead as AUS/FLUS, FN/SFN, or SFM, referred as indeterminate thyroid nodules, which therefore require diagnostic resections (lobectomy or total thyroidectomy) for further histopathological examination. Of indeterminate thyroid nodules, an approximate 20% will be found as malignant after diagnostic resections,^4,5^ indicating the other 80% of patients will have undergone an unnecessary surgical operation. As for patients with ND FNA biopsies that contain inadequate or insufficient follicular cells for standard cytological diagnosis, additional FNA biopsy is required to repeat the cytological evaluation, resulting in delays in diagnosis and increases of stress and cost to these patients. However, patients whose nodules persistently remain ND are referred for diagnostic surgery, and approximately 12% of nodules will be found to have malignant histopathological characteristics.^3,5^ Molecular assay has been evolving as an ancillary approach to assist in identifying malignant neoplasms in preoperative FNA specimens.^3,6^ A broad range of tests using gene panels have shown promising performance in diagnostic accuracy, including Afirma Gene Expression Classifier,^7,8^ genomic sequencing classifier,^9^ and ThyroSeq v2 or v3,^10,11,12^ but there is currently no single unequivocal test available for definitive assessment of thyroid nodules.^13^ In addition, these tests are mostly accessible in limited test centers and require obtaining additional fresh FNA biopsies. A molecular assay able to use the residual tissue from routine indeterminate FNA biopsies or ND FNA specimens in a diagnostic test to reveal genetic anomalies, in parallel with the FNA cytological examination results on the same batch of FNA biopsies, could not only enhance a more definitive cytological interpretation but also redefine the clinical value of the residual FNA samples, which are considered discard after cytological examination. Such a test would likely spare these patients from the need for a repeat FNA biopsy or diagnostic operation.

*BRAF* (OMIM 164757) variations are detected in 26% to 84% of papillary thyroid cancers (PTCs)^14,15,16,17^ and 10% to 15% of all human cancers.^8,18,19^ The most prevalent type of *BRAF* variation is BRAF V600E, in which an amino acid valine (V) was substituted to glutamic acid (E) at codon 600 (p.V600E) caused by transversion T to A at nucleotide 1799 (c. T1799A) in exon 15 of the *BRAF* gene.^18,19,20^ The prevalence of BRAF V600E in PTC varies significantly among geographic regions, from a relatively low rate (29%-69%) in the US and Europe^21,22^ to a high rate (76.4%) in East Asia.^23^ It has also been differentially detected in different variants of PTC, with an approximate 45% to 75.3% of classic variant PTC (cPTC), 100% of tall cell variant, and 12% to 40% of follicular variant,^16,21,22^ and nearly 10% to 24% of anaplastic thyroid cancers (ATC).^17,24^ However, BRAF V600E has not been detected in follicular carcinomas, medullary thyroid carcinomas, noninvasive follicular thyroid neoplasms with papillary-like nuclear features (NIFTP), or benign thyroid nodules.^15,24^ The highly specific BRAF V600E variation, associated with poor clinicopathologic outcomes,^25,26^ is an early genetic event in PTC^6^; therefore, the accurate detection of BRAF V600E status in indeterminate thyroid nodules is clinically useful in identifying patients for targeted treatment regardless of histological type.^27,28,29^

The current methods for BRAF V600E detection have evolved from Sanger sequencing to immunohistochemistry (IHC) and next-generation sequencing (NGS) assays with different levels of sensitivity.^20^ These assays cannot accurately predict BRAF V600E status or assist in clinical decision-making when the sensitivity is low. In addition, the utility of the existing assays for identification of BRAF V600E in residual thyroid FNA samples has not been established. Recently, the digital polymerase chain reaction (dPCR) technique has emerged as a more sensitive method compared with Sanger sequencing or NGS for BRAF V600E detection.^30,31^ Locked nucleic acid (LNA) modification has been shown to improve structural stability and increase hybridization melt temperature^32,33,34^; hence, LNA-modified probes can improve the discrimination of single nucleotide mismatch and the specific rare variation detection in challenging samples (eg, formalin-fixed, paraffin-embedded [FFPE] tissue and biofluids) compared with traditional PCR probes. This study is aimed to develop a molecular assay to accurately detect BRAF V600E variation and quantify the variant allele fraction (VAF) in residual tissue from routine thyroid FNA biopsies using droplet dPCR based on the LNA probes.

## Methods

This diagnostic study was approved by the Sinai Health System Research Ethics Board. All patient data were deidentified. All participants provided written informed consent. Results are presented in accordance with the Standards for Reporting of Diagnostic Accuracy (STARD) reporting guidelines.

### Patient Specimens and Residual Tissue From Thyroid FNA Biopsies

This diagnostic study was performed at Sinai Health System, Mount Sinai Hospital, a University of Toronto–affiliated hospital and a primary referral center for patients with thyroid disorders in Toronto, Canada, from February 2019 to May 2021. A total of 271 patient samples were collected and divided into a validation set, containing 77 FFPE tissue specimens from thyroid tumors resected between January 2010 and February 2019, and a test set, containing 146 residual tissue specimens from consecutive ultrasonography-guided FNA biopsies with a follow-up of 48 matched surgical specimens collected from February 2019 to April 2021. The final histological diagnosis was made in accordance with World Health Organization^35,36^ and College of American Pathologists.^37^ The residual samples from routine thyroid FNA biopsies were collected from the Cytopathology Laboratory in Mount Sinai Hospital 2 weeks after the cytological examination was conducted according to the Bethesda System for Reporting Thyroid Cytopathology,^3^ and these remaining samples (referred to as *residuals* or *leftovers*) were considered discard.

### Development of Molecular Assay by Droplet dPCR Using LNA Probes

Genomic DNA extraction from follicular thyroid cancer–derived cell line FTC-133 harboring wild-type *BRAF*, PTC-derived BCPAP harboring variant BRAF V600E, FFPE specimens, and remaining tissue of thyroid FNA biopsies are described in detail in the eMethods in the Supplement. The droplet dPCR for *BRAF* variation detection was performed on the QX200 AutoDG Droplet Digital PCR System (Bio-Rad Laboratories), per the manufacturer’s protocol with minor modification^38^ in Central Scientific Laboratory of Lunenfeld-Tanenbaum Research Institute. To achieve optimal assay performance, primer sets BRAF98 and BRAF91, which generate amplicons at 98 bp and 91 bp in lengths covering nucleotide 1799 in exon 15 of the *BRAF* gene, and LNA specific probes for *BRAF* (T1799A) variation (labeled by 6-fluorescein amidite [6-FAM]) and wild-type *BRAF* (labeled by hexachloro-fluorescein [HEX]) incorporating 6 LNA monomers were designed and synthesized (Integrated DNA Technologies). The dPCR assays of wild-type *BRAF* and variant *BRAF* (T1799A) were performed in a 22-μL reaction mixture containing 11 μL dPCR Supermix for Probes (2×, no dUTP) (Bio-Rad Laboratories), 1.1 μL of primer set (20×), 1.1 μL of probes (20×) containing *BRAF* variant and wild-type LNA probes, 5 μL of genomic DNA, and 3.8 μL of deionized water. The *BRAF* variant allele and wild-type allele concentration in the final PCR reaction mix, presented as copies per microliter, were calculated from the values of 6-FAM-positive droplets (variant) and HEX-positive droplets (wild-type). Variant *BRAF* (T1799A)-positive (BCPAP DNA), wild-type *BRAF* (FTC-133 DNA), and blank control (H_2_O) were included in each assay to verify the assay condition and exclude potential contamination. Droplet generation, PCR amplification, droplet read and calculation, BRAF V600E IHC for validation, and assessment of specificity, sensitivity, and positive and negative predictive values are described in the eMethods in the Supplement.

### Statistical Analysis

Data were summarized as frequencies and percentages for categorical variables, and means and SDs for normally distributed continuous variables. Differences in distributions between the clinicopathological characteristics and molecular status were compared using 2-sided Fisher exact test for categorial variables and *t* test or analysis of variance test for parametric continuous measures. The degree of agreement between a pair of variables was assessed by linear regression and Cohen κ coefficient. SPSS statistical software version 22.0 (IBM Corporation) was used for all analyses. *P* values were 2-sided, and *P* < .05 was considered statistically significant.

## Results

### Baseline Characteristics of Tumor Specimens

A total of 271 tumor specimens were collected from 223 patients (mean [SD] age, 53.8 [15.3] years; 174 women [78.0%]; 49 [22.0%] men). The validation set contained 77 FFPE surgical specimens diagnosed as 36 follicular variants of PTC (FVPTC) (46.8%), 14 cPTCs (18.2%), 4 benign nodules (5.2%), and 23 NIFTPs (29.9%) (eTable 1 in the Supplement). The test set contained 146 specimens of remaining tissue from routine FNA biopsies, including 58 AUS/FLUS (39.7%), 11 SFM (7.5%), 27 malignant (18.5%), and 50 ND FNAs (34.2%) (eTable 2 in the Supplement).

### Molecular Assay for Specific Detection of BRAF V600E by LNA Probe–Based dPCR

**Figure 1.**
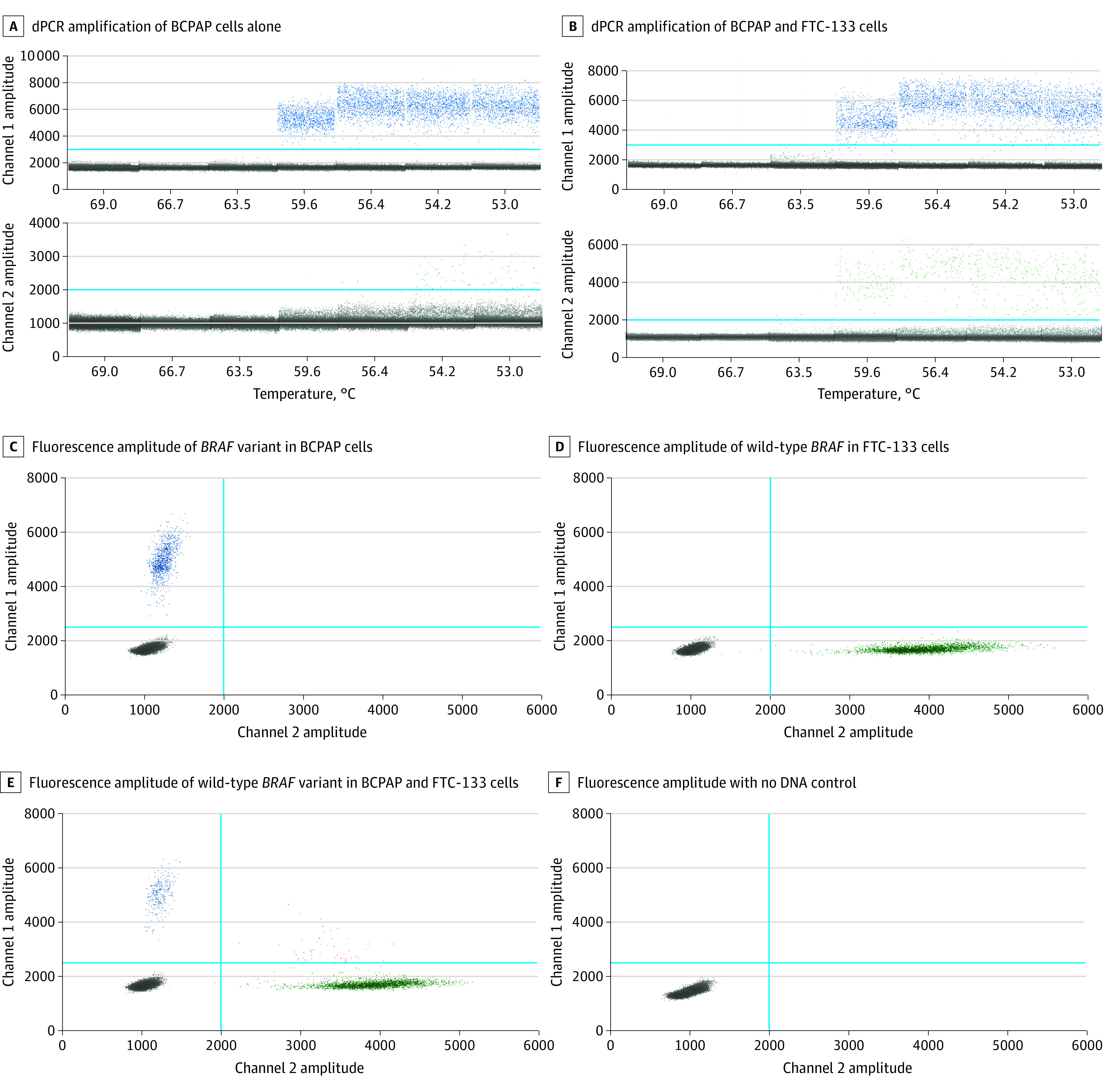
Establishment of Droplet Digital Polymerase Chain Reaction (dPCR) Detection of BRAF V600E Variation Using Locked Nucleic Acid Probes A and B, 1-Dimensional fluorescence amplitude plots showed all accepted droplets per well or group of wells in channel 1 and channel 2. The dPCR amplification of genomic DNA from BCPAP cells alone (A) or genomic DNA mix (at ratio 1:1) from BCPAP and FTC-133 cells (B) at gradient annealing temperatures from 53 °C to 69 °C using locked nucleic acid probes and primer set BRAF98. With threshold (blue line) established for positive and negative droplets in either channel 1 at 2500 or channel 2 at 2000, positive droplets in channel 1 (blue dots) indicate the specific binding of the locked nucleic acid 6-fluorescein amidite variant probes to *BRAF* (T1799A) and positive droplets in channel 2 (green dots) indicate the specific binding of locked nucleic acid hexachloro-fluorescein wild-type probes to wild-type *BRAF*, while negative droplets are displayed in gray. C-F, With thresholds across both channel 1 at 2500 and channel 2 at 2000, droplets were divided into distinct clusters separated by different colors. Negative droplets are displayed in gray, while positive droplets in channel 1 (blue dots) represent 6-fluorescein amidite signals (variant *BRAF*) and positive droplets in channel 2 (green dots) represent hexachloro-fluorescein signals (wild-type *BRAF*).

Droplet dPCR based on LNA probes successfully generated the positive droplets specific for wild-type *BRAF* and variant *BRAF* (T1799A) and yielded the optimal cluster tightness and separation at 59.6 °C using primer set BRAF98, whereas it failed to generate positive droplets at 63.5 °C or greater, or it generated nonspecific positive droplets for HEX at 56.4 °C or below (Figure 1A-B; eFigure 1 in the Supplement). Droplet dPCR failed to generate specific positive droplets at all tested temperatures using primer set BRAF91 (eFigure 2 in the Supplement). The variant (Figure 1C) and wild-type *BRAF* (Figure 1D) were specifically detected in genomic DNA isolated from BCPAP and FTC-133 cells, respectively. Double-positive droplets indicated the detection and quantification of the variant as well as wild-type *BRAF* alleles in mixtures of genomic DNA from both BCPAP and FTC-133 cells (Figure 1E). In contrast, the lack of positive droplets showed neither variant nor wild-type *BRAF* alleles were detected in the control reaction with no DNA template (Figure 1F). Therefore, the 6-FAM– and HEX-labeled LNA probes in combination with primer set BRAF98 were demonstrated to specifically detect and quantify the variant BRAF V600E and wild-type *BRAF* DNA respectively with optimal droplet segregation at an annealing temperature of 59.6 °C (Figure 1; eFigure 1 and eFigure 2 in the Supplement).

### Accuracy, Reproducibility, and Sensitivity of dPCR Assay for BRAF V600E

**Figure 2.**
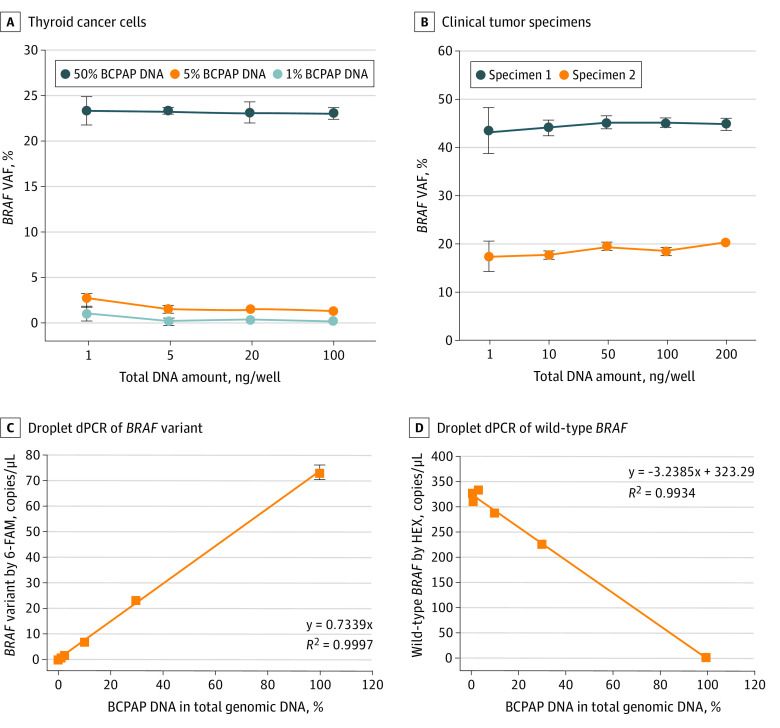
Linearity of Variant DNA Quantification Across a Broad Dynamic Range of Input DNA A and B, Reproducible detection and quantification of *BRAF* variant allele fraction (VAF) in thyroid cancer cell lines with a broad range of input DNA amount (A) and in 2 clinical tumor specimens with a wide range of input DNA amount (B). C and D, Droplet digital polymerase chain reaction (dPCR) quantification of fractional abundances of *BRAF* variant (C) and wild-type alleles (D) across a broad dynamic range of DNA dilutions. The detection was performed in 3 independent tests with triplicate wells for each sample. Dots indicate means; error bars, 95% CIs; 6-FAM, 6-fluorescein amidite; and HEX, hexachloro-fluorescein.

The accuracy and reproducibility in detection of BRAF V600E by droplet dPCR assay was demonstrated in a wide range of input DNA mixes (1 to 100 ng per well) via quantification of *BRAF* VAF at a mean (SD) of 0.50% (0.11%) at 1% dilution of BCPAP DNA in the background of FTC-133 DNA, 1.79% (0.16%) at 5% dilution, and 23.51 (0.28%) at 50% dilution (Figure 2A). In 2 tumor specimens, the mean (SD) VAFs were 45.51% (1.15%) and 19.20% (0.73%) in a wide range of input DNA (1 to 200 ng per well) (Figure 2B). Droplet dPCR assays detected variant *BRAF* (T1799A) in BCPAP DNA samples at all dilutions (ie, 0.3%, 1%, 3%, 10%, 30%, and 100%) in the background of FTC-133 DNA. The linearity of dPCR quantification across a broad dynamic range of *BRAF* variant or wild-type alleles (copies per microliter) was calculated as y = 0.7339x and *R*^2^ = 0.9996 for variant *BRAF* (T1799A) and y = −3.2385x +323.29 and *R*^2^ = 0.9934 for wild-type *BRAF* (Figure 2C and D). The analytical sensitivity presented as the lowest detectable variant DNA concentration was calculated as 0.02 copies/μL. The dPCR results were highly reproducible in various *BRAF* (T1799A) variation dilutions (eg, coefficient of variance in 0.3% DNA, 9.63%; coefficient of variance in 1.0% DNA, 7.41%). The specificity of the optimized dPCR assays was established by exclusive detection of variant *BRAF* DNA in BCPAP and wild-type *BRAF* DNA in FTC-133 cells and by accurate quantification of the *BRAF* VAF in various fractions of variant DNA.

### Detection of BRAF V600E in FFPE Tumor Tissue by dPCR Assay

**Figure 3.**
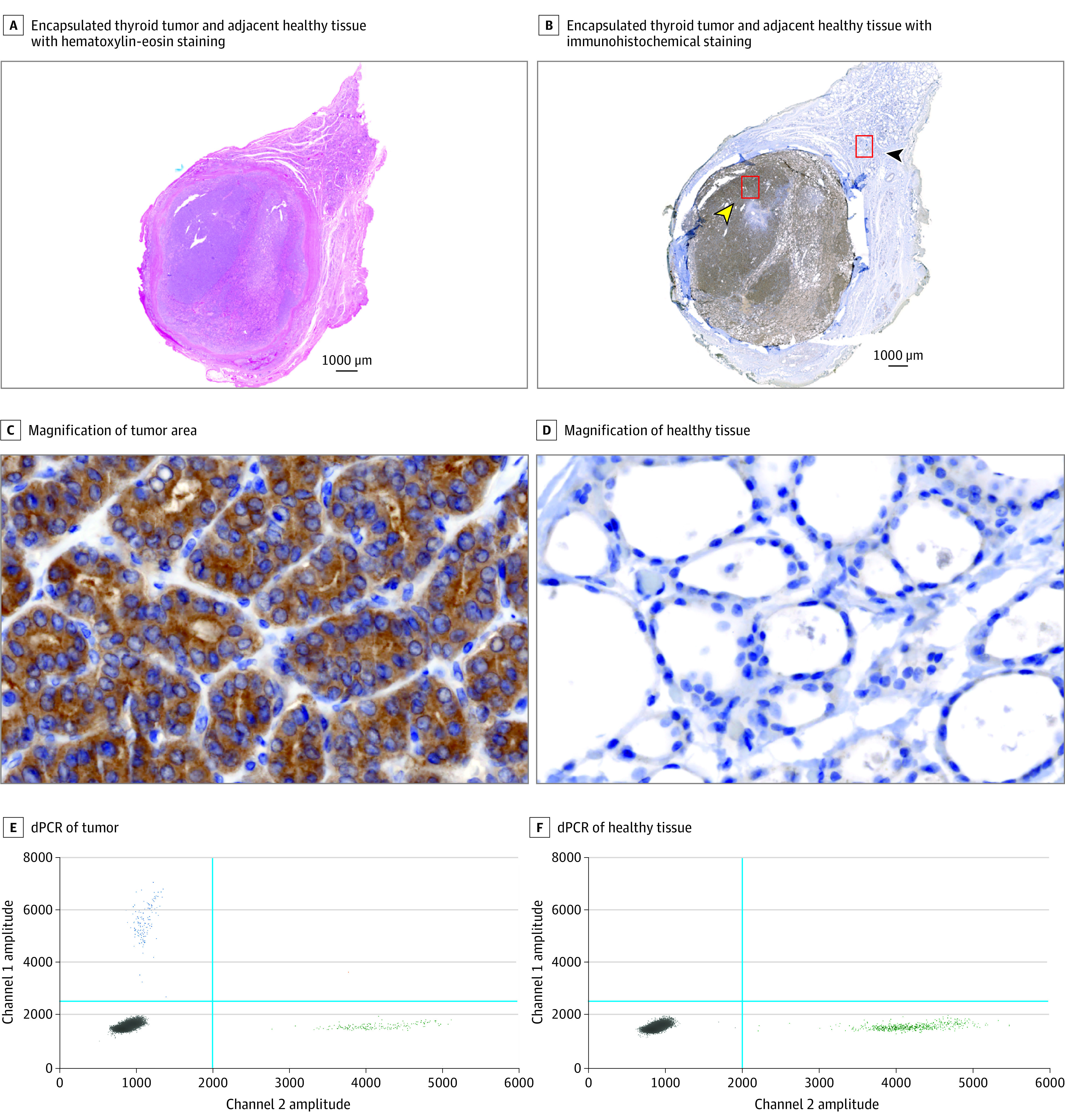
Immunohistochemistry (IHC) Staining and Digital Polymerase Chain Reaction (dPCR) Assay Detection of BRAF V600E Variation A-D, The presence of BRAF V600E was specifically stained in the cell cytoplasm within tumor area indicated by the yellow arrow (B) and shown at high magnification (C), but not in its adjacent healthy tissue indicated by the black arrow (B) and shown at high magnification (D). The amount of input DNA was 10 ng in each reaction of dPCR.

BRAF V600E IHC staining showed the variant BRAF protein was expressed exclusively in the cytoplasm of tumor cells but not in the adjacent healthy tissue (Figure 3A-D). Consistently, dPCR assay detected the presence of *BRAF* (T1799A) only in DNA from the tumor tissue but not from the adjacent healthy tissue (Figure 3E and F). In the validation test, BRAF V600E variation was detected in 16 of 77 FFPE tissue specimens (20.8%) by dPCR assay and 13 of 77 FFPE tissue specimens (16.9%) by IHC staining, with no significant association with patient demographics (Table 1). The VAF quantified by dPCR ranged from low positive, at 0.02%, to high positive, at 47.8%, in 16 *BRAF* variation–positive specimens, including 12 high positive specimens (>1% VAF) and 4 low positive specimens (<1% VAF). Of 12 specimens with greater than 1% VAF, including 6 of 14 cPTCs and 6 of 36 FVPTCs, 10 were stained unequivocally positive, while 1 was stained weakly and patchily and 1 was negative for BRAF V600E by IHC. Of 4 specimens with less than 1% VAF from FVPTCs, 2 were weakly and patchily stained in less than 10% of malignant cells, while the other 2 were negative by IHC (eTable 1 in the Supplement). Hence, VAF at 1% was used as a cutoff to classify high or low positive in the subsequent dPCR quantification. All *BRAF* variation–positive specimens were PTC, including encapsulated FVPTC (VAF, 25.1%), cPTC (VAF, 13.3%), and cPTC with extensive follicular growth (VAF, 37.9%), but none were benign or NIFTP tissues (eFigure 3 in the Supplement). Both methods consistently identified 13 specimens positive for BRAF V600E and 61 specimens negative for BRAF V600E, showing a perfect concordance (κ = 0.873; *P* < .001) in revealing *BRAF* status (estimated IHC score = 0.173 × (*BRAF* VAF[%]) + 0.230; *R*^2^ = 0.79; *P* < .001) (eFigure 4 in the Supplement). The dPCR assay detected BRAF V600E in all 13 specimens found positive in IHC staining as well as in 3 specimens found negative in IHC staining, exhibiting a 100% specificity and a sensitivity 32.0% (95% CI, 19.1%-44.9%) in identifying PTCs, with an improvement of 23.08% compared with IHC staining, at a sensitivity 26.0% (95% CI, 13.1%-38.9%).

**Table 1.** Demographic and Clinicopathological Characteristics of Patients in the Validation Set Assessed by dPCR Assay and IHC Staining

Characteristic	Patients, No. (%)			*P* value^a^
	Total (n = 77)	Nonmalignant tumor (n = 27)	Malignant tumor (n = 50)	
Sex				
Women	59 (76.6)	19 (32.2)	40 (67.8)	.40
Men	18 (23.4)	8 (44.4)	10 (55.6)	
Age at diagnosis, y				
Mean (SD)	50.3 (13.6)	52.6 (13.9)	49.0 (13.6)	.27
<45	35 (45.5)	11(31.4)	24 (68.6)	.63
≥45	42 (54.5)	16 (4.8)	26 (61.9)	
Thyroidectomy^b^				
Total	66 (88.3)	21 (31.8)	45 (68.2)	.06
Partial	9 (11.7)	6 (66.7)	3 (33.3)	
Tumor size, mean (SD), cm	2.86 (1.71)	2.91 (2.06)	2.83 (1.52)	.84
IHC staining, BRAF V600E status				
Negative	64 (83.1)	27 (42.2)	37 (57.8)	.003
Positive	13 (16.9)	0	13 (100)	
dPCR assay, BRAF V600E status				
Negative	61 (79.2)	27(44.3)	34 (55.7)	.001
Positive	16 (20.8)	0	16 (100)	

Abbreviations: dPCR, digital polymerase chain reaction testing; IHC, immunohistochemical staining.

^a^ Fisher exact test (2-sided) for categorial variables and *t* test for independent parametric continuous measures.

^b^ Missing data for 2 patients owing to incomplete information.

### Detection of BRAF V600E Variation in the Residual Tissue From FNA Biopsies

The DNA isolated from each residual thyroid FNA biopsy yielded a mean (SD) of 0.87 (1.08) μg with no significant difference among groups of AUS/FLUS, SFM, malignant, and ND FNA (eTable 2 in the Supplement). The dPCR assay detected BRAF V600E variation in 39 of 146 remaining FNA specimens (26.7%) and *BRAF* wild-type allele in 107 of 146 remaining FNA specimens (73.3%), with no significant association with patient demographics (Table 2; eTable 2 in the Supplement). The 39 BRAF V600E–positive specimens included 12 of 27 malignant findings (44.4%), 4 of 11 SFM findings (36.4%), 14 of 58 AUS/FLUS findings (24.1%), and 9 of 50 ND findings (18.0%) and showed a VAF ranging from low positive, at 0.02%, to high positive, at 71.80%. At short-term follow-up (mean [range], 91 [3-223] days), 48 patients underwent resection, including 14 of 39 patients(35.9%) with the BRAF V600E variant and 34 of 107 patients (31.8%) without the BRAF V600E variant, according to residual FNA analysis (Table 2). At the thyroidectomy, among 14 patients with the BRAF V600E variant according to residual FNA analysis, 13 were diagnosed with PTC and 1 was diagnosed with ATC; while among 34 patients without the BRAF V600E variant, 24 were diagnosed with PTC, 1 was diagnosed with metastatic renal cell carcinoma invasion to the thyroid, 1 was diagnosed with squamous cell carcinoma invasion to the thyroid, and 8 were diagnosed with benign tumors (Table 2). *BRAF* status of the matched surgical tumor tissue showed a high concordance with that of residual FNA samples (κ = 0.789; *P* < .001) (eFigure 5 in the Supplement), except 1 patient with FLUS, whose residual FNA biopsy tissue was negative for the BRAF V600E variant but whose surgical specimen was positive, and 2 patients with SFM and 1 patient with malignant PTC, whose residual FNA biopsies were positive for the BRAF V600E variant tissue but whose surgical specimens were negative. In the overall malignancy analysis based on final histopathological findings, the specificity was 100%, sensitivity was 35.0% (95% CI, 22.1%-47.9%), positive predictive value was 100%, and negative predictive value was 23.5% (95% CI, 11.1%-36.0%) for dPCR assays of *BRAF* variation on residual FNA samples. Based on the *BRAF* status identified in surgical tumor tissue, dPCR assays of residual FNA samples showed the specificity of 91.7% (95% CI, 91.67%-91.67%), sensitivity of 91.7% (95% CI, 78.7%-104.6%), positive predictive value of 78.6% (95% CI, 78.6%-78.6%), and negative predictive value of 97.1% (95% CI, 84.6%-109.5%) to predict *BRAF* variation.

**Table 2.** BRAF V600E Status Detected in Indeterminate FNAs and the Matched Surgical Tumor Specimens

Characteristics	Patients, No. (%)			*P* value^a^
	Total (n = 146)	Residual FNA biopsy findings		
		BRAF V600E positive (n = 39)	BRAF V600E negative (n = 107)	
**All patients**				
Sex				
Women	115 (78.8)	32 (27.8)	83 (72.2)	.65
Men	31 (21.2)	7 (22.6)	24 (77.4)	
Age at diagnosis, y				
Mean (SD)	55.7 (15.8)	54.6 (16.2)	56.0 (15.8)	.63
<45	31 (21.2)	8 (25.8)	23 (74.2)	>.99
≥45	115(78.8)	31 (27.0)	84 (73.0)	
Source of biopsies				
Left side	60 (41.1)	18 (30.0)	42 (70.0)	.83
Right side	81 (54.5)	20 (24.7)	61 (75.3)	
Isthmus	5 (3.4)	1 (20.0)	4 (80.0)	
FNA cytological findings				
ND	50 (34.2)	9 (18.0)	41 (82.0)	.07
AUS/FLUS	58 (39.7)	14 (24.1)	44 (75.9)	
SFM	11 (7.5)	4 (36.4)	7 (63.6)	
Malignant	27 (18.5)	12 (44.4)	15 (55.6)	
**Patients with surgical specimens**				
No. (%)	48 (100)	14 (29.2)	34 (70.8)	
Thyroidectomy				
Total	21 (43.8)	6 (28.6)	15 (71.4)	>.99
Partial	27 (56.2)	8 (29.6)	19 (70.4)	
Tumor size, mean (SD), cm^b^	2.20 (1.53)	1.65 (1.27)	2.49 (1.61)	.11
FNA cytological findings				
ND	9 (18.8)	0	9 (100)	.09
AUS/FLUS	13 (27.1)	3 (23.1)	10 (76.9)	
SFM	10 (20.8)	4 (40.0)	6 (60.0)	
Malignant	16 (33.3)	7 (43.7)	9 (56.3)	
Histopathological findings				
Benign	8 (16.7)	0	8 (100)	.09
Papillary thyroid cancer	37 (77.1)	13 (35.1)	24 (64.9)	
Anaplastic thyroid cancer	1 (2.1)	1 (100)	0	
Other malignant tumor^c^	2 (4.2)	0	2 (100)	
*BRAF* status in surgical tissue				
Positive	12 (25.0)	11 (91.7)	1 (8.3)	<.001
Negative	36 (75.0)	3 (8.3)	33 (91.7)	

Abbreviations: AUS/FLUS, atypia of undetermined significance or follicular atypia of undetermined significance; ND, insufficient for diagnosis; SFM, suspicious for malignancy.

^a^ Fisher exact test (2-sided) for categorical variables and *t* test for independent parametric continuous measures.

^b^ Result was computed based upon information from 37 PTC and 1 ATC tumors.

^c^ One patient was diagnosed with squamous cell carcinoma, and 1 patient was diagnosed with metastatic renal cell carcinoma that invaded to thyroid.

## Discussion

In this diagnostic study, a sensitive and specific molecular diagnostic assay using LNA probe–based droplet dPCR was established for detection and quantification of BRAF V600E variation in the remaining tissue of routine thyroid FNA biopsies by amplifying a 98 bp amplicon and forming well-separated, specific, and tight droplet clusters. The clinical utility of dPCR assay for BRAF V600E detection was validated by BRAF V600E IHC staining of FFPE specimens and further tested in residual FNA biopsies. Our data showed the clinical utility of residual samples from routine FNA biopsies in identifying genomic alterations in thyroid nodules by a sensitive molecular assay, providing deep molecular profiles to assist in cytological diagnosis in the same batch of cytological biopsies.

BRAF V600E is an important oncogenic variation and an attractive therapeutic target in a variety of human cancers, such as thyroid cancer, melanoma, colon cancer, ovarian cancer, and non–small cell lung cancer.^20,26,39,40,41^ Timely management of these cancers requires a rapid, cost-effective, and accurate assay for BRAF V600E detection. Sequencing of tumor DNA, such as Sanger sequencing and pyro-sequencing, is a common method for identifying *BRAF* variations. However, the sensitivity of Sanger sequencing is largely influenced by the rate of malignant cells in a tumor. Approximately 6.6% to 20% of variant alleles could be identified in a background of wild-type alleles.^20,39,42^ Real-time PCR analysis using traditional sequence-specific probes is not reliable for detection of variation less than 10%.^43^ NGS assay allows high-throughput identification of tumor-related genetic alterations of different genes, including *BRAF* variations. NGS panels have become increasingly used in an attempt to stratify the risk of malignancy in all types of advanced tumors via a 1-size-fits-all test,^9,10,11,13,44^ although their clinical impact on tumor management is currently insubstantial.^13,45^ IHC staining with specific monoclonal antibodies is characterized as a rapid and relatively inexpensive assay for BRAF V600E variation.^46,47^ IHC is an attractive and alternative approach to PCR and Sanger sequencing for surgical specimens because it allows direct visualization of BRAF V600E expression at a single-cell resolution, especially when cancer cells are admixed with excess nonneoplastic follicular cells, stromal cells, or lymphocytic thyroiditis.^48,49,50^ In this study, the molecular assay by dPCR enabled sensitive and specific detection of BRAF V600E variation by incorporating the use of LNA technology, which improved the discrimination of single nucleotide mismatch compared with traditional real-time PCR probes.^32,33,34^ In our validation test, IHC using Clone VE1 antibodies showed homogenous BRAF V600E staining in the cytoplasm within tumor cells on the positively stained sections, but the dPCR assay exhibited an improved sensitivity in detecting the presence of BRAF V600E. In addition, quantification of *BRAF* VAF by dPCR assay eliminated issues commonly present in IHC, such as discrepancies in staining procedures among different platforms^8,48^ and discord in interpretation of the staining results.^8,48,51,52^ Of note, IHC is of limited value in biopsy specimens, since equivocal staining results frequently occur on direct smears or cell blocks with scant tumor cellularity or purity.^49,52,53,54^ In this study, *BRAF* variation was successfully identified in 26.7% of residual FNA biopsies. Follow-up of 48 patients who underwent surgical resection found good correlation of the dPCR assay of *BRAF* status between the residual FNA tissue and the matched surgical specimens. In 1 patient, the *BRAF* variation was detected in surgical tissue but the wild-type allele was predicted in residual FNA samples, possibly because of an inadequate sampling of PTC cells via FNA biopsy. The 3 patients with residual FNA results positive for BRAF V600E variation at a low level but whose surgical specimens were negative for *BRAF* variation indicated that when the variation occurred in rare cells in a tumor and the sampling of resected tissue lacked variant cells, analysis of such surgical specimens could not correctly reveal the *BRAF* status in the tumor.

The accurate diagnosis of indeterminate FNAs is still a challenge to the clinician treating patients with thyroid disease. This study demonstrated that the residual tissue from routine FNA biopsies could be clinically useful for molecular diagnostic assays. Regardless of whether the residual tissues from FNA biopsies are freshly prepared or stored in CytoLyte up to 6 months, DNA extracted from the remaining tissue can be used for the LNA probe–based dPCR assays. The utility of residual FNA biopsies makes it possible to evaluate molecular and cytological profiles of thyroid nodules simultaneously on the same FNA biopsy. Molecular analysis of BRAF V600E status or other genetic alterations on the residual tissues of thyroid FNA biopsies, especially for indeterminate or ND FNA results, could assist in reaching a more definitive cytological diagnosis. The preoperative cytological identification of *BRAF* variant–positive PTC could guide the initial surgical treatment and subsequent therapeutic decision-making.^29^ In addition, molecular assay on the residual FNA biopsies would be cost-effective in identifying well-characterized variations because it reduces the cost and burden to patients by sparing them from a repeat thyroid FNA biopsy procedure, while most other tests require an additional FNA sample or surgical specimens. Importantly, this novel dPCR assay could be extended to identify BRAF V600E status in biopsies from other types of cancer, such as melanoma, non–small cell lung cancer, colon cancer, colorectal cancer, and ovarian cancer, in the context of precision medicine.

### Limitations

This study has some limitations. First, the molecular assay by dPCR is restricted to detection of a single target: BRAF V600E variant. Complementary assays on other clinically actionable oncogenes or variations are needed to stratify malignancy for tumors that do not have BRAF V600E. Second, the accuracy of VAF quantification is still influenced by the rate of malignant cells in a specimen, and false-negative results could occur when biopsies were sampled from tumor-adjacent healthy tissue. Third, we were not able to identify an association of the *BRAF* VAF in the residuals of FNA biopsies with histopathological status in the current cohort. The molecular assay by dPCR may be further validated via analysis of *BRAF* VAF and follow-up of clinicopathological outcomes using larger cohorts from multiple clinic centers.

## Conclusions

In this diagnostic study, a convenient and cost-effective molecular assay using LNA probe–based droplet dPCR was developed to quantitatively detect BRAF V600E variation in the residual tissue of routine thyroid FNA biopsies. Given the high sensitivity and accuracy in detection of *BRAF* variation when analyzing a trace amount of tumor biopsies, this newer molecular assay could be implemented in most hospitals or clinical centers for timely identification and monitoring of *BRAF* variation status in tumors, thereby improving the treatment of patients with cancer harboring BRAF V600E.

## Notes

Supplement.
eMethods.
**eTable 1.** Demographic and Clinicopathological Features of Patients and BRAF V600E in Thyroid PTC by Droplet dPCR Assay and IHC Staining **eTable 2.** BRAF V600E Variation Status in Indeterminate FNAs With Demographics and Clinicopathological Features **eFigure 1.** Optimization of Droplet dPCR Conditions **eFigure 2.** Droplet dPCR Based on LNA Probes Failed in Specifically Detecting BRAF V600E Variation Using Primer Set BRAF91 **eFigure 3.** BRAF V600E Variation in Surgical FFPE Tissue Was Concordantly Detected by Both IHC Staining and dPCR Assay **eFigure 4.** Scatter Diagram of IHC and dPCR With Regression Line **eFigure 5.** 2-Dimensional Plots of BRAF V600E Variation in the Leftover of FNA Samples and Matched Surgical Specimens by dPCR
