# Errors in Abstract and Results

**DOI:** 10.1001/jamanetworkopen.2022.5698

**Published:** 2022-03-15

In the Original Investigation titled “Development of a Molecular Assay for Detection and Quantification of the *BRAF* Variation in Residual Tissue From Thyroid Nodule Fine-Needle Aspiration Biopsy Specimens,”^1^ published October 6, 2021, there were errors in the 95% CI values in the Abstract and Results sections. This article has been corrected.^1^
