# Supplementary material for: Development of a Molecular Assay for Detection and Quantification of the BRAF Variation in Residual Tissue From Thyroid Nodule Fine-Needle Aspiration Biopsy Specimens
Source: JAMA Netw Open. 2021 Oct 6;4(10):e2127243. doi: 10.1001/jamanetworkopen.2021.27243 (PMC8495535; doi:10.1001/jamanetworkopen.2021.27243)
Supplement: Supplement. — eMethods. eTable 1. Demographic and Clinicopathological Features of Patients and BRAF V600E in Thyroid PTC by Droplet dPCR Assay and IHC Staining eTable 2. BRAF V600E Variation Status in Indeterminate FNAs With Demographics and Clinicopathological Features eFigure 1. Optimization of Droplet dPCR Conditions eFigure 2. Droplet dPCR Based on LNA Probes Failed in Specifically Detecting BRAF V600E Variation Using Primer Set BRAF91 eFigure 3. BRAF V600E Variation in Surgical FFPE Tissue Was Concordantly Detected by Both IHC Staining and dPCR Assay eFigure 4. Scatter Diagram of IHC and dPCR With Regression Line eFigure 5. 2-Dimensional Plots of BRAF V600E Variation in the Leftover of FNA Samples and Matched Surgical Specimens by dPCR [file jamanetwopen-e2127243-s001.pdf]

Supplemental Online Content

Fu G, Chazen RS, MacMillan C, Witterick IJ. Development of a molecular assay for detection and quantification of the *BRAF* variation in residual tissue from thyroid nodule fine-needle aspiration biopsy specimens. *JAMA Netw Open*. 2021;4(10):e2127243. doi:10.1001/jamanetworkopen.2021.27243

eMethods.

eTable 1. Demographic and Clinicopathological Features of Patients and BRAF V600E in Thyroid PTC by Droplet dPCR Assay and IHC Staining

eTable 2. BRAF V600E Variation Status in Indeterminate FNAs With Demographics and Clinicopathological Features

eFigure 1. Optimization of Droplet dPCR Conditions

eFigure 2. Droplet dPCR Based on LNA Probes Failed in Specifically Detecting BRAF V600E Variation Using Primer Set BRAF91

eFigure 3. BRAF V600E Variation in Surgical FFPE Tissue Was Concordantly Detected by Both IHC Staining and dPCR Assay

eFigure 4. Scatter Diagram of IHC and dPCR With Regression Line

eFigure 5. 2-Dimensional Plots of BRAF V600E Variation in the Leftover of FNA Samples and Matched Surgical Specimens by dPCR

This supplemental material has been provided by the authors to give readers additional information about their work.

© 2021 Fu G et al. *JAMA Network Open.*

eMethods.

1. Thyroid Cancer Cells Harboring Wild-type and Variant BRAF

Thyroid cancer cell lines FTC-133 and BCPAP[1], respectively harboring wild-type BRAF and variant BRAF V600E (kindly provided by Dr. Shilpa Thakur, NIH, MD, US), were used for extraction of genomic DNA as the negative and positive controls for verifying the detection of BRAF variations. FTC-133 cells were cultured in DMEM growth medium (Thermo Fisher Scientific) supplemented with 10% fetal bovine serum (FBS) and BCPAP cells were cultured in RPMI 1640 medium (Thermo Fisher Scientific) with 5% FBS, each containing 100 U/mL penicillin and 100 mg/mL streptomycin in a humidified 5% CO_2 incubator. The cell authentication of two cell lines originated from human thyroid follicular (FTC-133) and papillary cancers (BCPAP) were confirmed by short tandem repeat profiling analysis by TCAG Genetic Analysis Facility (The Centre for Applied Genomics, Toronto, CAN).

2. Genomic DNA Extraction from Thyroid Cancer Cells, FFPE and FNA Biopsies

Monolayer FTC-133 or BCPAP cell culture were detached at 90% confluence, then fixed in CytoLyte solution overnight at room temperature to mimic the procedure of preparation of FNA biopsies. FTC-133 or BCPAP cells and the remaining material of FNA biopsies preserved in CytoLyte solution were pelleted first and re-suspended in 200 µl of PBS for DNA isolation using the DNeasy Blood & Tissue Kits (Qiagen, Hilden, Germany), according to manufacturer's instructions. FFPE tissue blocks were sectioned at 10-µm thickness, and DNA extraction was performed with QIAamp DNA FFPE Tissue Kit (Qiagen, Hilden, Germany). All patient samples were de-identified. The DNA isolation and subsequent testing were all blinded to the patients'

© 2021 Fu G et al. *JAMA Network Open.*

demographics, diagnosis, and disease status. The extracted DNA elute was stored at -20°C. DNA concentration was measured using NanoDrop 8000 (Thermo Fisher Scientific).

3 Immunohistochemistry for the BRAF V600E Variation

Immunohistochemistry (IHC) staining was performed using FFPE tissue sections (4 μm) and the anti-BRAF ^{V600E} (VE1) antibodies (Roche Diagnostics, ON, CAN) on the Ventana Benchmark Ultra (Ventana Medical Systems, AZ, USA) with Cell Conditioning 1 for 64 min, pre-peroxidase inhibition and primary antibody incubation for 16 min at 37°C according to the manufacturer's procedures in Mount Sinai Services (MSH, ON, CAN). The OptiView Universal DAB IHC Detection Kit (Ventana Medical Systems) was used to detect BRAF V600E expression on the Ventana Benchmark Ultra autoimmunostainer[3]. Tissues were manually counterstained with CAT Hematoxylin and Bluing Reagent (Biocare Medical, CA, USA) for 2 min. PTC tissues harboring the BRAF V600E variation and tissues lacking BRAF V600E variation identified by direct Sanger sequencing, were included in the staining procedure in every batch. Stained slides were scanned by Microscopy Slide Scanner ZEISS Axio Scan.Z1 (Zeiss, Oberkochen, Germany) in the OPTical IMAging (OPTIMA) Facility at Lunenfeld-Tanenbaum Research Institute in Mount Sinai Hospital and all H&E or immunostaining images were acquired via ZEISS ZEN (blue edition). Slides were evaluated by an experienced pathologist who was blinded to the BRAF mutational status. BRAF V600E immunostaining was scored by combining percentage scale and overall intensity level of cells staining positive[4]. The percentage scale was determined by the percentage of cells staining positive in cytoplasm according to: 0 (≤ 10%), 1 (11–30%), 2 (31–50%), 3 (51–70%), or 4 (> 70%), and the intensity level of stained cells was graded as 1= weak or focal cytoplasmic staining, 2 = moderate or 3 = strong. The total score value ranges from 0 to

© 2021 Fu G et al. *JAMA Network Open.*

7. Equivocal or no staining in tumor cells and non-specific staining of colloids were considered negative.

4. **Assessment of Sensitivity, Specificity, and Positive and Negative Predictive Values**

The accuracy and reproducibility of dPCR detection of BRAF V600E was further assessed using serial dilutions of DNA samples from FTC-133 cells mixed with 1%, 5% and 10% of DNA from BCPAP cells, respectively, and serial dilutions of DNA samples from clinical specimens. *BRAF* VAF was calculated by *BRAF* variant alleles (copies) vs. the total copies of *BRAF* variant alleles and *BRAF* wild-type alleles in each reaction from all triplicate wells. The analytical specificity was validated with a temperature gradient to ensure 6-FAM LNA probe to specifically detect *BRAF* variant alleles and HEX LNA probe specifically detecting *BRAF* wild-type alleles, as well as each probe showing no cross-reactivity between variant and wild-type clusters using FTC-133 and BCPAP DNA by 2-dimensional plot analysis. The optimal annealing temperature is determined by the condition in which the variant 6-FAM probe exhibits no false positives in the wild-type FTC-133 DNA samples and relative distance between the 6-FAM-only and HEX-only clusters is maximal. The specificity was further confirmed by Sanger sequencing of PCR amplified products (Eurofins Genomics, KY, USA). To assess the detection sensitivity, dPCR reactions were performed with 50 ng of input DNA at serial dilutions (ie. 0, 0.3%, 1%, 3%, 10%, 30%, and 100%) of DNA from *BRAF* variant BCPAP cells in the background of *BRAF* wild-type DNA from FTC-133 cells. For example, the 0.3% mix contained 0.3% of BCPAP DNA and 99.7% of FTC-133 DNA. Sensitivity was determined by the limit of detection (LoD), the analytical lowest level, at which the variant allele DNA could be reliably distinguished from a blank (zero concentration) sample (LoB)[2]. A serial of varying concentrations of BRAF variant

© 2021 Fu G et al. *JAMA Network Open.*

alleles (copies/ul) vs. seral dilutions of input variant DNA (%) was achieved to determine the model of a linear calibration curve as well as the LoB and LoD. The LoB was estimated using the following formula: LoB = $Mean_{blank}$ + 1.645*(SD_{blank}). The $Mean_{blank}$ and SD_{blank} are calculated using replicates, up to 20 wells of a zero calibrator or blank sample (0% variant DNA and 100% wild-type DNA). The LoD was calculated by analyzing triplicates from three separate experiments and using the following formula: LoD = LoB + 1.645*($SD_{0.3\% \text{ sample}}$). The reproducibility was expressed as coefficient of variance (CV) of standard deviation of BRAF V600E detection result. The CV was quantified using the following formula: CV = SD/Mean. The clinical sensitivity, specificity, and positive and negative predictive values with 95% confidence intervals of the residual FNA-assessed *BRAF* variation status for tumor malignancy were calculated using standard formulas in accordance with STARD 2015.

Availability of Methods and Materials

Further information and requests for dPCR assays should be directed to and will be fulfilled by the Lead Contact, Guodong Fu (David.Fu@sinaihealth.ca; gdfu2002@gmail.com).

References

1. Thakur S, Daley B, Gaskins K, et al. Metformin targets Mitochondrial Glycerophosphate Dehydrogenase (mGPDH) to control Rate of Oxidative Phosphorylation and growth of thyroid cancer in vitro and in vivo. *Clinical cancer research : an official journal of the American Association for Cancer Research.* 2018.
2. Armbruster DA, Pry T. Limit of blank, limit of detection and limit of quantitation. *Clin Biochem Rev.* 2008;29 Suppl 1:S49-52.
3. Dvorak K, Aggeler B, Palting J, McKelvie P, Ruszkiewicz A, Waring P. Immunohistochemistry with the anti-BRAF V600E (VE1) antibody: impact of pre-analytical conditions and concordance with DNA sequencing in colorectal and papillary thyroid carcinoma. *Pathology.* 2014;46(6):509-517.
4. Fu G, Polyakova O, MacMillan C, Ralhan R, Walfish PG. Programmed Death - Ligand 1 Expression Distinguishes Invasive Encapsulated Follicular Variant of Papillary Thyroid Carcinoma from Noninvasive Follicular Thyroid Neoplasm with Papillary-like Nuclear Features. *EBioMedicine.* 2017;18:50-55.

© 2021 Fu G et al. *JAMA Network Open.*

eTable 1. Demographic and Clinicopathological Features of Patients and BRAF V600E in Thyroid PTC by Droplet dPCR Assay and IHC Staining

**Characteristics**	**Patient, No. (%)**					***P* value** [b]
	**Total** (n = 77)	**Benign** (n = 4)	**NIFTP** (n = 23)	**FVPTC** (n = 36)	**cPTC** (n = 14)	
**Sex**						*.547*
Women	59 (76.6)	2 (3.4)	17 (28.8)	29 (49.2)	11 (18.6)	
Men	18 (23.4)	2 (11.1)	6 (33.3)	7 (38.9)	3 (16.7)	
**Age Mean (SD), y**	50.3 (13.6)	53.8 (15.9)	52.4 (13.9)	49.1(14.6)	49.0 (10.3)	*.750*
**Age category, y**						*.775*
< 45	35 (45.5)	2 (5.7)	9 (25.7)	16 (45.7)	8 (22.9)	
≥ 45	42 (54.5)	2 (4.8)	14 (33.3)	20 (47.6)	6 (14.3)	
**Thyroidectomy**[a]						*.003*
Total	66 (88.0)	2 (3.0)	19 (28.8)	34 (51.5)	11 (16.7)	
Partial	9 (22.0)	2 (22.2)	4 (44.4)	0	3 (33.3)	
**Tumor size Mean (SD), cm**	2.86 (1.71)	2.60 (2.99)	2.97 (1.94)	2.76 (1.42)	3.02 (1.78)	*.935*
**Encapsulated**[a]						*<.001*
No	7 (9.3)	2 (28.6)	0	0	5 (71.4)	
Yes	68 (90.7)	0	23 (33.8)	36 (52.9)	9 (13.2)	
**BRAF V600E IHC**						*<.001*
Score = 0	64 (83.1)	4 (6.2)	23 (35.9)	29 (45.3)	8 (12.5)	
Score < 3	3 (3.9)	0	0	3 (100)	0 (0)	
Score ≥ 3	10 (13.0)	0	0	4 (40.0)	6 (60.0)	
**BRAF V600E dPCR**						*.005*
0 VAF	61 (79.2)	4 (6.6)	23 (37.7)	26 (42.6)	8 (13.1)	
< 1% VAF	4 (5.2)	0	0	4 (100)	0	
≥ 1% VAF	12 (15.6)	0	0	6 (50.0)	6 (50.0)	

Abbreviations: cPTC: classical variant papillary thyroid cancer; FVPTC: follicular variant papillary thyroid cancer; NIFTP: non-invasive follicular thyroid neoplasm with papillary-like nuclear features; VAF: Variant allele fraction.
[a] Missing data for 2 patients owning to incomplete information.
[b] Fisher's exact test (2-sided) for categorial variables and ANOVA test for independent parametric continuous measures.

© 2021 Fu G et al. *JAMA Network Open.*

eTable 2. BRAF V600E Variation Status in Indeterminate FNAs with Demographics and Clinicopathological Features

Characteristics	Patient, No. (%)					*P value*[*]
	**Total** (n = 146)	**ND** (n = 50)	**AUS/FLUS** (n = 58)	**SFM** (n = 11)	**Malignant** (n = 27)	
**Sex**						*.167*
Women	115 (78.8)	40 (34.8)	48 (41.7)	10 (8.7)	17 (14.8)	
Men	31 (21.2)	10 (32.3)	10 (32.3)	1 (3.2)	10 (32.3)	
**Age Mean (SD), y**	55.7 (15.8)	58.7 (15.4)	54.9 (16.1)	54.7 (15.2)	52.0 (15.8)	*.320*
**Age category, y**						*.261*
< 45	31 (21.2)	7 (22.6)	13 (41.9)	2 (6.5)	9 (29.0)	
≥ 45	115 (78.8)	43 (37.4)	45 (39.1)	9 (7.8)	18 (15.7)	
**Source of biopsies**						*.899*
Left side	60 (41.1)	21 (35.0)	24 (40.0)	3 (5.0)	12 (20.0)	
Right side	81 (55.5)	27 (33.3)	31 (38.3)	8 (9.9)	15 (18.5)	
Isthmus	5 (3.4)	2 (40.0)	3 (60.0)	0	0	
**DNA yield Mean (SD), µg**	0.87(1.08)	0.81(1.26)	0.97 (1.09)	0.91(0.77)	0.74 (0.75)	*.803*
**BRAF VAF category**						*.010*
0 VAF	107 (73.3)	41 (38.3)	44 (41.1)	7 (6.5)	15 (14.0)	
< 1% VAF	21 (14.4)	4 (19.0)	11 (52.4)	3 (14.3)	3 (14.3)	
≥ 1% VAF	18 (12.3)	5 (27.8)	3 (16.7)	1 (5.6)	9 (50.0)	

Abbreviations: ND, Insufficient for diagnosis; AUS/FLUS, Atypia of undetermined significance/ Follicular atypia of undetermined significance; SFM, Suspicious for malignancy; VAF, Variant allele fraction.
[a] 2-sided Fisher exact test for categorial variables and ANOVA test for parametric continuous measures.

© 2021 Fu G et al. *JAMA Network Open.*

eFigure 1. Optimization of Droplet dPCR Conditions

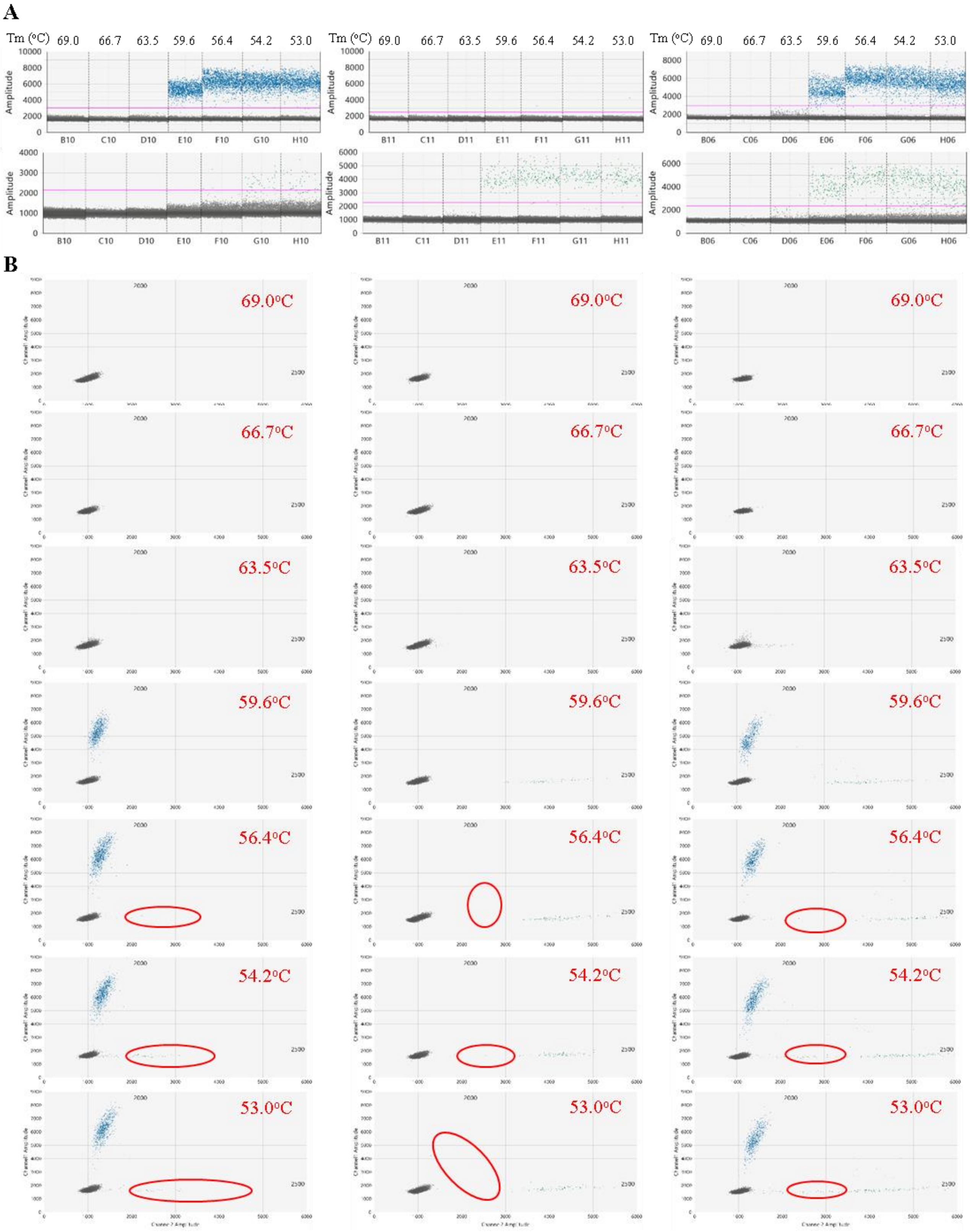

© 2021 Fu G et al. *JAMA Network Open.*

1-dimensional (**A**) and two-dimensional plots (**B**) of droplet dPCR were shown using BRAF98/ LNA probes at gradient temperatures (53^oC-69^oC) with 50ng of BCPAP DNA alone (left column), 50ng of FTC-133 DNA (middle column) or mix of 50ng of BCPAP and FTC-133 DNA each (right column) in A&B panels. Red circles marked nonspecific positive droplets at 53^oC-56.4^oC in panel B. In 2-Dimensional plot, droplets were divided into distinct clusters separated by blue dots for 6-FAM positive droplets, green dots for HEX positive droplets and grey dots for negative droplets with thresholds across both channel 1 at 2500 and channel 2 at 2000.

© 2021 Fu G et al. *JAMA Network Open.*

eFigure 2. Droplet dPCR Based on LNA Probes Failed in Specifically Detecting BRAF V600E Variation Using Primer Set BRAF91

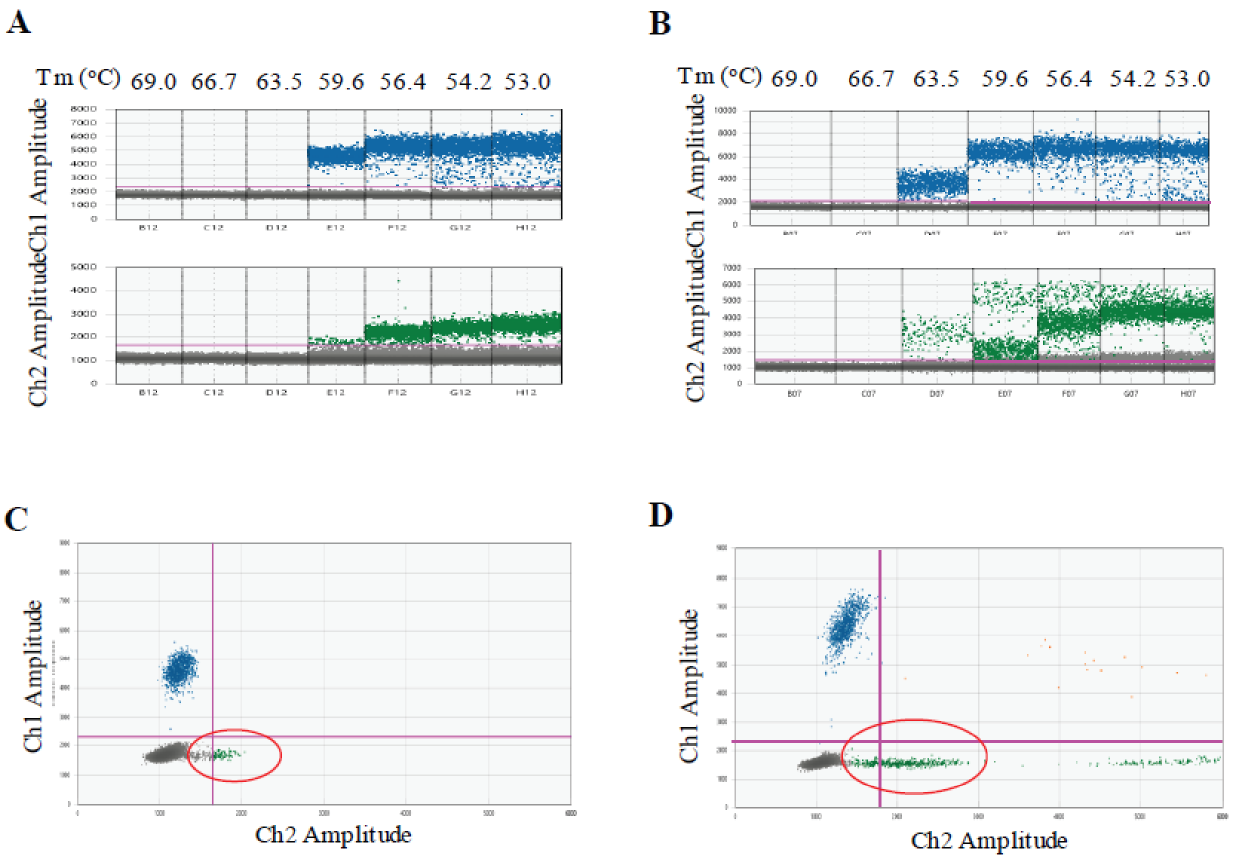

A and B. 1-dimensional plot showed droplet dPCR amplification of genomic DNA of BCPAP cell alone (**A**) or genomic DNA mix (at ratio 1:1) from BCPAP and FTC-133 cells (**B**) at gradient annealing temperatures from 53°C to 69°C using primer set BRAF91/LNA probes. Blue dots indicated signal from the specific binding of the LNA FAM variant probes to BRAF V600E (in upper panels of A and B) and green dots indicated signal from the specific binding of LNA HEX wild-type probes to wild-type BRAF (in lower panels of A&B), while negative droplets are displayed in grey dots. **C and D**, 2-dimensional plot of droplet dPCR using primer set BRAF91/LNA probes at 59.6°C with 50ng of BCPAP DNA alone (**C**) or mix of 50ng of BCPAP and FTC-133 DNA each (**D**). Non-specific amplified droplets were marked in red circle. In 2-Dimensional plot, droplets were divided into distinct clusters separated by blue dots for 6-FAM positive droplets, green dots for HEX positive droplets and grey dots for negative droplets with thresholds across both channel 1 at 2500 and channel 2 at 2000.

© 2021 Fu G et al. *JAMA Network Open.*

eFigure 3. BRAF V600E Variation in Surgical FFPE Tissue Was Concordantly Detected by Both IHC Staining and dPCR Assay

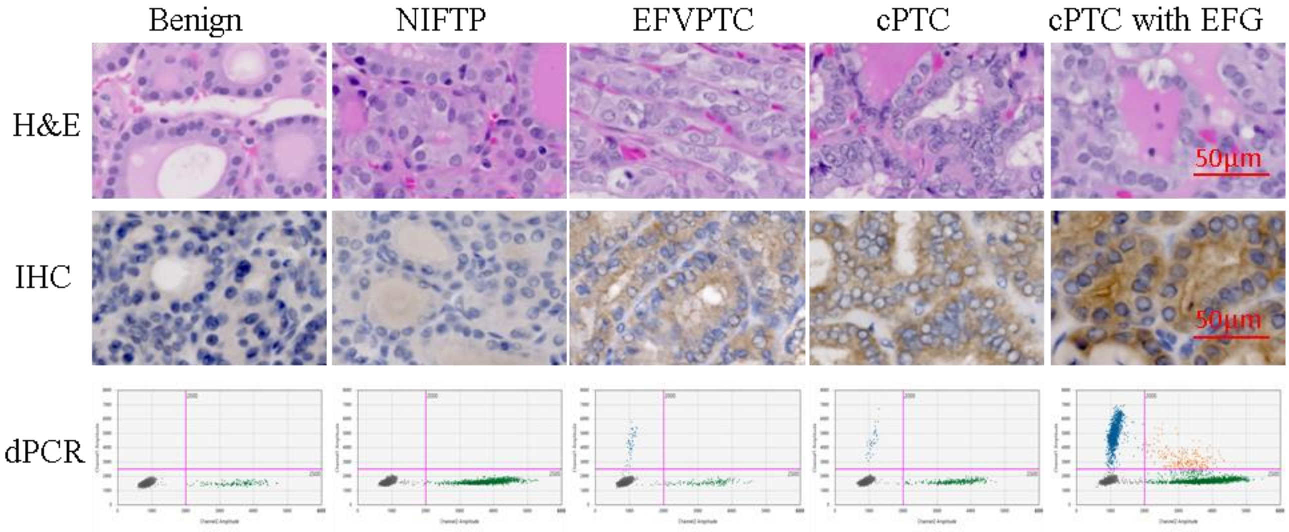

IHC and dPCR analyses of BRAF V600E variation in subtypes of PTC, benign and NIFTP tissue with a correlation between BRAF VAF and BRAF V600E staining. Top panel (H&E) demonstrated morphological features of examined tumors, and middle and bottom panels showed results of BRAF IHC staining and dPCR assay, respectively. The *BRAF* VAF quantified by dPCR assays was 0 in benign, 0 in NIFTP, 25.1% in EFVPTC, 13.3% in cPTC and 37.9% cPTC with EFG. In 2-Dimensional plot, droplets were divided into distinct clusters separated by blue dots for 6-FAM positive droplets, green dots for HEX positive droplets and grey dots for negative droplets with thresholds across both channel 1 at 2500 and channel 2 at 2000. Abbreviations: VAF, Variant allele fraction; NIFTP, non-invasive follicular thyroid neoplasm with papillary-like nuclear features; EFVPTC, encapsulated follicular variant papillary thyroid cancer; cPTC, classic variant papillary thyroid cancer; and EFG, extensive follicular growth.

© 2021 Fu G et al. *JAMA Network Open.*

eFigure 4. Scatter Diagram of IHC and dPCR with Regression Line

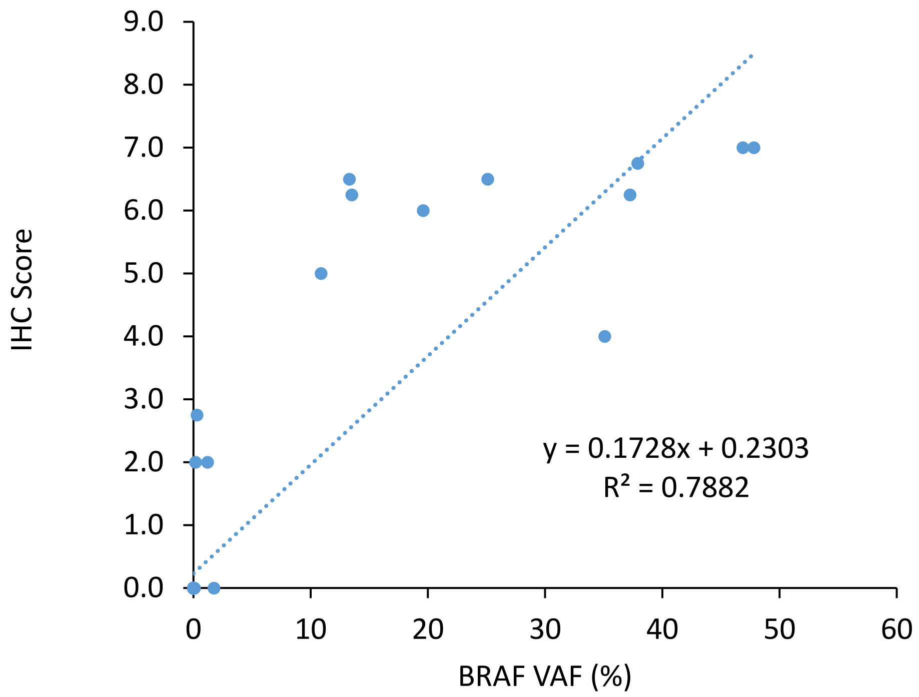

BRAF variant allele fraction (VAF) (%) Line Fit Plot showed the *BRAF* variation status: Predicted IHC score = 0.173 x *BRAF* VAF (%) + 0.230; R^2 = 0.79; p <.0001, with a Pearson Correlation = 0.90, p <.001.

© 2021 Fu G et al. *JAMA Network Open.*

eFigure 5. 2-Dimensional Plot of BRAF V600E Variation in the Residuals of FNA Samples and Matched Surgical Specimens by dPCR

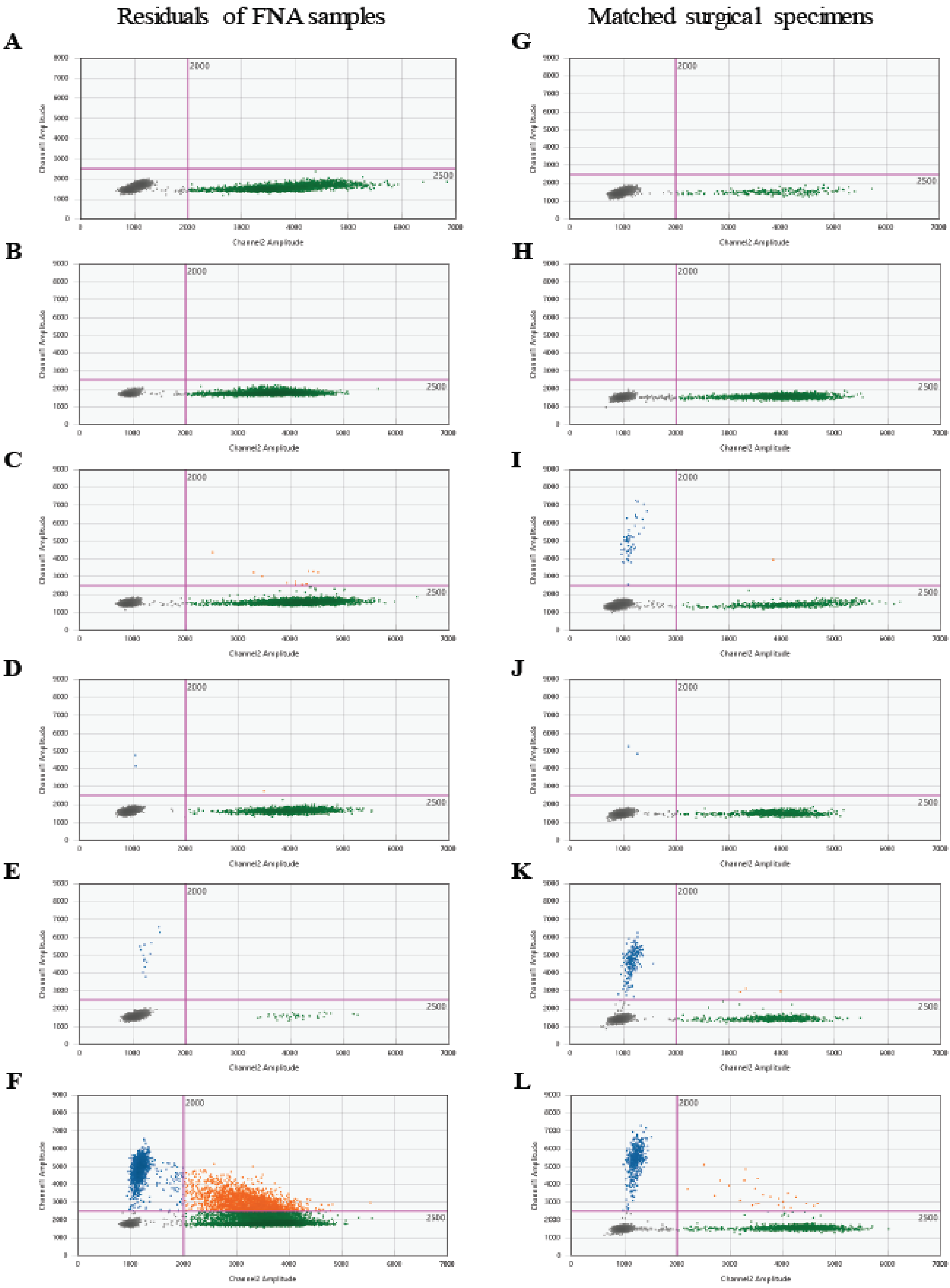

© 2021 Fu G et al. *JAMA Network Open.*

The left panel of **A-F** demonstrated dPCR detection of BRAF V600E variation in the residual tissue of FNA samples and quantification of *BRAF* VAF at 0 in benign FNA residual tissue (**A**), 0 in ND FNA residual tissue (**B**), 0.14% in AUS FNA residual tissue (**C**), 0.13% in FLUS FNA residual tissue (**D**), 25.9% in SFM FNA residual tissue (**E**) and 18.6% in malignant FNA residual tissue (**F**). The right panel of **G-L** demonstrated dPCR detection of BRAF V600E variation in the matched surgical specimens and quantification of *BRAF* VAF at 0 in benign matched surgical specimens (**G**), 0 in ND matched surgical specimens (**H**), 7.92%in AUS matched surgical specimens (**I**), 0.20% in FLUS matched surgical specimens (**J**), 22.8% in SFM matched surgical specimens (**K**) and 24.2% in malignant matched surgical specimens (**L**). The residual of benign FNA samples (**A**) and its matched surgical specimens (**G**) were included as control samples in the test. In 2-Dimensional plot, droplets were divided into distinct clusters separated by blue dots for 6-FAM positive droplets, green dots for HEX positive droplets and grey dots for negative droplets with thresholds across both channel 1 at 2500 and channel 2 at 2000. Abbreviations: VAF, Variant allele fraction; ND, Insufficient for diagnosis; AUS/FLUS, Atypia of undetermined significance/ Follicular atypia of undetermined significance; SFM, Suspicious for malignancy.

© 2021 Fu G et al. *JAMA Network Open.*
